# Leveraging Cross-Subject Transfer Learning and Signal Augmentation for Enhanced RGB Color Decoding from EEG Data

**DOI:** 10.3390/brainsci16020195

**Published:** 2026-02-06

**Authors:** Metin Kerem Öztürk, Dilek Göksel Duru

**Affiliations:** 1 Department of Computer Science, Faculty of Engineering, Turkish-German University, Istanbul 34820, Türkiye; kerem@keremozturk.net; 2 Department of Molecular Biotechnology, Faculty of Science, Turkish-German University, Istanbul 34820, Türkiye

**Keywords:** color classification, EEG decoding, transfer learning, deep learning

## Abstract

Objectives: Decoding neural patterns for RGB colors from electroencephalography (EEG) signals is an important step towards advancing the use of visual features as input for brain–computer interfaces (BCIs). This study aims to overcome challenges such as inter-subject variability and limited data availability by investigating whether transfer learning and signal augmentation can improve decoding performance. Methods: This research introduces an approach that combines transfer learning for cross-subject information transfer and data augmentation to increase representational diversity in order to improve RGB color classification from EEG data. Deep learning models, including CNN-based DeepConvNet (DCN) and Adaptive Temporal Convolutional Network (ATCNet) using the attention mechanism, were pre-trained on subjects with representative brain responses and fine-tuned on target subjects to parse individual differences. Signal augmentation techniques such as frequency slice recombination and Gaussian noise addition improved model generalization by enriching the training dataset. Results: The combined methodology yielded a classification accuracy of 83.5% for all subjects on the EEG dataset of 31 previously studied subjects. Conclusions: The improved accuracy and reduced variability underscore the effectiveness of transfer learning and signal augmentation in addressing data sparsity and variability, offering promising implications for EEG-based classification and BCI applications.

## 1. Introduction

Electroencephalography (EEG) is a non-invasive technique for recording brain activity that is widely used to investigate the electrical response of the brain at the scalp in response to human environmental and mental events, including in the study of visual perception. Decoding these neural signals has the potential to improve brain–computer interfaces (BCIs) and has potential applications such as assisting individuals with disabilities by interpreting visual stimuli directly from brain activity [1]. In many traditional visual BCI paradigms such as P300 and SSVEP-based systems, command selection is typically achieved through gaze-dependent visual attention [2], with the system aiming to decode the color of the presented visual stimulus from spatial–temporal patterns of low-frequency EEG activity recorded at the scalp level [3]. Visual stimuli can provide a greater variety of control signals in a shorter period of time compared to other stimuli, such as sound [4]. Recently, several studies have focused on discriminating EEG signals recorded in response to different hue values [5,6], various shapes and colors [7,8,9,10,11], rapid-event visual stimuli [12,13], and chromaticity presented at different luminance levels [14,15]. These studies demonstrate that brain electrical activity changes as a function of the color distinctions in the visual stimuli. Differences in EEG dynamics across subjects lead to inconsistent neural responses to identical stimuli, while temporal variability within the same subject further affects the classification performance across sessions [16,17]. Recent BCI literature shows that transfer learning and domain selection methods can substantially alleviate the effects of both inter-subject and sessional variability [18,19,20,21,22], and has demonstrated the significance of TL on prediction rate for multiclass EEG classification through the implementation of a compact convolutional neural network (CNN) architecture for EEG-based BCI [23].

This research investigates the reliability of EEG signals in classifying colors, overcoming challenges such as inter-subject variability and limited data availability. From a methodological perspective, numerous studies have investigated EEG-based color decoding, employing different analytical techniques and success metrics. For example,. Ludvigsen et al. (2021) [24] used time-frequency analysis paired with a Riemannian classifier (FgMDM) on the same dataset of 31 subjects as Fløtaker et al. (2023) [25], which is also used in this study. After applying certain quality criteria, 23 subjects remained and the study achieved an average classification accuracy of 74.4% for red, green, and blue (RGB) visual evoked potentials (VEPs). Similarly, Fløtaker et al. [25] applied different deep learning architectures on both electrode space and source space with the same dataset of 31 subjects. The best result was obtained with DeepConvNet on the electrode space. They reported an average classification accuracy of 77% for RGB color classification and a peak accuracy of 96% for the best performing subject. In contrast, Hajonides et al. (2021) [8] adopted a different perspective by using Linear Discriminant Analysis (LDA) to decode parametric color variations from EEG signals on a dataset of 30 participants. This study presented 48 equally spaced tones in CIELAB color space as bilateral Gabor gratings and demonstrated reliable decoding of color-specific neural patterns over 200 ms windows. Success was measured in terms of the consistency and discriminability of these patterns across subjects and trials rather than reporting accuracy.

Other relevant studies have generally worked with smaller datasets or fewer trials, which limit their generalizability and are therefore not discussed here.

To generate more reliable inputs for BCIs by improving classification performance, we propose a methodology that combines transfer learning and signal augmentation with deep learning architectures, namely, DeepConvNet and ATCNet. This approach aims to address inter-subject variability and data scarcity, with a general performance evaluation based on all 31 subjects in the dataset. This enables a more detailed and focused discussion of the work of Ludvigsen et al. (2021) [24], who used only 23 subjects.

The remainder of this paper is structured as follows: Section 2 describes the dataset used in the study and the processing methods applied to it, followed by a detailed explanation of the training and classification procedures. The classification results are presented in Section 3, and the implications and limitations of the findings are discussed in Section 4.

## 2. Materials and Methods

### 2.1. Dataset

The dataset utilized in this study is described in [24]. It comprises 60-channel EEG recordings obtained during a color perception experiment complemented by structural magnetic resonance imaging (MRI) scans of 31 participants. Ten of the participants were female, with a mean age of 28.9 years (standard deviation = 7.4 years). In addition, 27 participants were right-handed. All participants had normal or near-normal corrected vision and did not exhibit color vision deficits.

**Figure 1:**
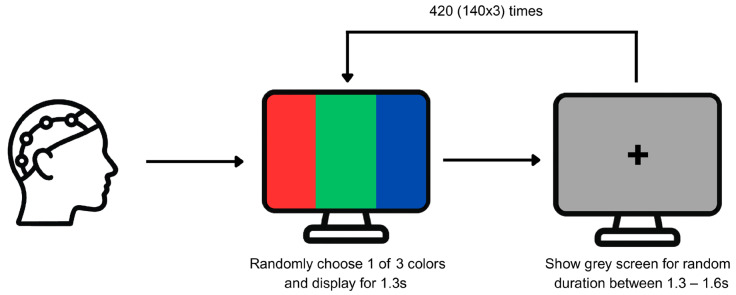
Schematic depiction of the data recording process.

The experimental design involved presenting various color stimuli to participants seated in front of a screen, as depicted in Figure 1. Each of the colors red, green, and blue was presented 140 times each in a random order and for 1.3 s each. Each stimulus was followed by a gray screen showing a central fixation cross for a randomly selected interval ranging from 1.3 to 1.6 s to attenuate neural adaptation. The colors were presented in full-screen format and the fixation cross appeared only during the gray screen intervals to maintain consistent eye focus across participants [25].

Data recording was conducted at the Aalto NeuroImaging Facility at Aalto University in Finland, in a sophisticated room with three-layer magnetic shielding [25]. A wet electrode system with 64 channels was used to record the EEG. Of these, sixty channels were used to capture EEG signals on the scalp, while the remaining four channels were responsible for recording electrooculography (EOG) to observe eye movements [24].

### 2.2. Signal Preprocessing

**Figure 2:**
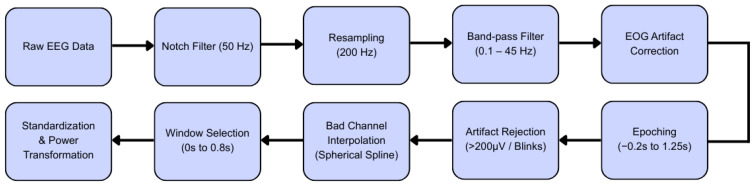
Schematic representation of the EEG signal preprocessing pipeline, detailing the sequential stages from raw data acquisition to final feature transformation for deep learning analysis.

The purpose of pre-processing EEG signals is to bring the raw signals into a clean and artifact-free form, ready to be used in future analyses and deep learning modeling. The Python (3.12.8) libraries MNE (1.9.0) and scikit-learn (1.6.0) were used to implement the methods detailed in this section. We used the same adopted processing pipeline used in [25], with a few basic modifications (Figure 2). First, we applied a Notch filter at 50 Hz to eliminate power line interference [26]. The data was then resampled to 200 Hz to reduce computational demands while maintaining the fundamental frequency content and removing high-frequency noise [27]. A band-pass filter was then used to isolate frequencies between 0.1 Hz and 45 Hz, focusing on the EEG signals of interest while ignoring low-frequency shifts and high-frequency noise [28]. To address ocular artifacts, blink events were identified on the dedicated EOG channels using the MNE ‘find_eog_events’ algorithm, and Signal Space Projection (SSP) was calculated and integrated into the data to reduce their influence. Stimulus events were removed from the stimulation channel, and the continuous data were divided into epochs ranging from −0.2 s to 1.25 s relative to each stimulus onset, and epochs exceeding 200 µV in EEG amplitude were discarded to remove significant artifacts, resulting in an average of 14 discarded trials per subject. In an additional step, epochs containing blinks occurring within 0.1 s before and within 0.1 s after the event occurred were excluded from the training data to further ensure data quality. Epochs were extracted as a 3D sequence (trials, channels, samples) and data from between 0 s and 0.8 s post-stimulus were selected for training to target the most relevant time window. Model performance was improved by standardizing the data and subsequently applying the Yeo–Johnson power transformation, where the optimal transformation parameter for each feature was estimated via Maximum Likelihood Estimation (MLE) to achieve a Gaussian-like distribution., the Yeo–Johnson transformation was fitted exclusively on the training data within each fold of the cross-validation process and then applied to the validation and test sets using the parameters derived from the training fold.

#### Interpolation of Bad Channels

In this study, transfer learning, which will be covered in detail in Section 2.3, was used to adapt a pre-trained baseline model to individual subjects, requiring a consistent input size across all subjects. To achieve this, interpolation of bad channels is favored rather than excluding them, as it preserves the uniformity of the data structure by maintaining the same number of channels for each subject. With this approach, the same base model becomes feasible for all participants. The identification of bad channels relied on the pre-existing annotations provided within the original dataset. Across the entire cohort, an average of 0.42 EEG channels per subject were identified as bad; for the subset of subjects that required interpolation, the average was 2.17 channels per subject.

The bad EEG channels were interpolated using the spherical spline method [29] available in the Python MNE library. This technique maps sensor locations to a unit sphere and estimates the signal at faulty sensor locations by utilizing data from surrounding functional sensors. By considering the spatial arrangement of the electrodes, it effectively reconstructs the missing signals and preserves the quality of the EEG dataset [30].

**Figure 3:**
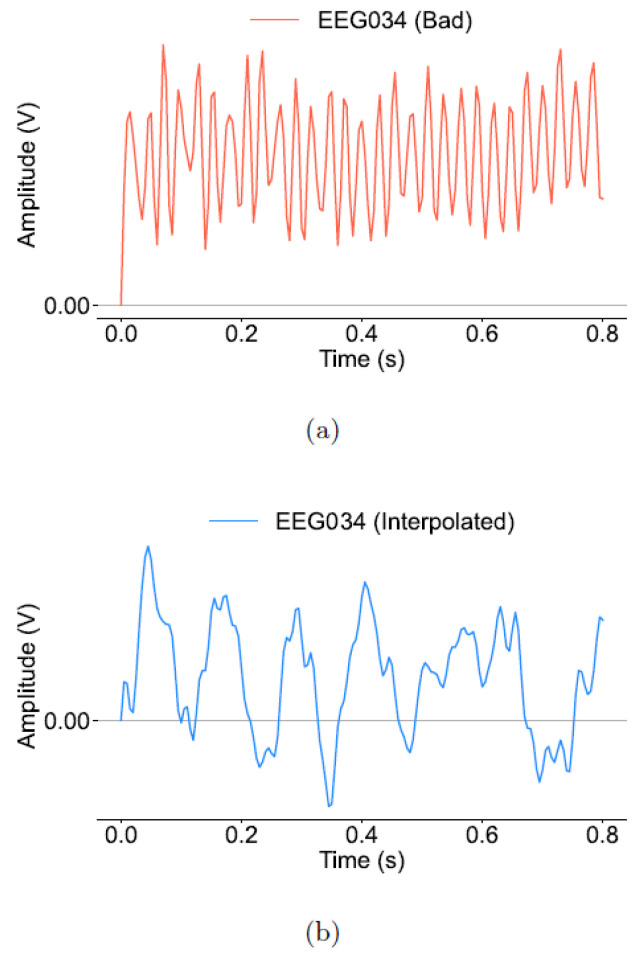
(**a**) EEG signal from the Oz channel of subject 1 for the 0.0 s to 0.8 s interval marked as “bad” exhibiting noticeable artifacts; (**b**) interpolated signal demonstrating a smoother, more continuous waveform.

Figure 3 shows a comparison between a bad channel and its interpolated counterpart, illustrating the success of this approach. This underlines the ability of the spherical spline approach to produce a reasonable signal approximation and supports the reliability of the processed data for further analysis.

### 2.3. Transfer Learning

In this study, transfer learning was used to improve within-subject classifier performance by utilizing information from subjects with high-quality data and representative brain responses. EEG signals vary significantly between individuals, which makes cross-subject generalization difficult [11,31]. Transfer learning has been shown to improve performance by exploiting shared feature patterns across subjects [32]. This approach is particularly beneficial with limited or low-quality data. This decision was motivated by the observation of inconsistent classification accuracies across subjects when using different deep neural network (DNN) architectures such as DeepConvNet. For example, the highest accuracy for one subject reached 96%, while the lowest accuracy was only 42%. In other words, the model is basically capable of analyzing this data. Rather than attributing these differences solely to the model design, we hypothesized that data-related factors, such as the number and quality of trials, played a larger role. This conclusion is supported by the low accuracy of most of the eight subjects who were excluded from the first study because they did not meet the inclusion criteria.

Statistical analysis identified channels with consistent inter-subject correlations in three conditions (condition 0: red; condition 1: green; condition 2: blue). For each condition, pairwise Pearson correlations of mean EEG responses across subjects were calculated per channel. A one-sample *t*-test with Bonferroni correction for multiple comparisons (*α* = 0.05) was used to test whether these correlations were significantly positive [33]. The results showed that most channels had significant correlations, specifically, 55 for condition 0 (red), 49 for condition 1 (green), and 53 for condition 2 (blue). However, five channels (1, 2, 3, 35, and 37) did not show significant correlations in all conditions, possibly indicating subject-specific information or noise. These channels were retained as their relevance may differ between subjects due to differences in electrode sensitivity. Adaptive weighting of DNNs is expected to manage these subject-specific differences according to their importance on the outcome [34].

**Figure 4:**
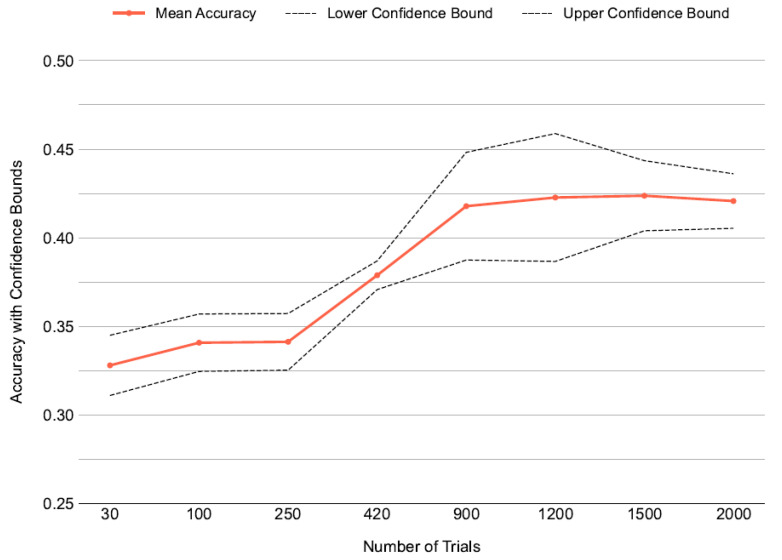
Mean test accuracy of DeepConvNet vs. number of training trials (30–2000), randomly sampled from all subjects; dashed lines show confidence bounds from 5-fold cross-validation.

Next, we carried out a cross-validation experiment to determine the minimum number of trials needed to train DeepConvNet effectively. Training trial numbers were varied (30, 100, 250, 420, 900, 1200, 1500, 2000) using combined data from multiple subjects (excluding a fixed test subject, subject 1) with class-balanced sampling. Performance was assessed on the test subject’s data, averaged over five folds. Figure 4 shows accuracy rising from 0.32 at 30 trials to 0.38 at 420 trials, then stabilizing between 0.42 and 0.43 beyond that, with slight gains up to 2000 trials. Confidence bounds tightened with more trials, suggesting greater reliability. The same method was applied separately to both DeepConvNet and ATCNet, and accuracy and confidence intervals were evaluated. These findings demonstrated that using 3 subjects for DeepConvNet and 2 subjects for ATCNet is optimal for training the baseline model to be used in transfer learning.

To optimize transfer learning, subjects with brain responses most representative of the population were selected using inter-subject correlations. Pairwise correlations of the average EEG responses were calculated for each condition and averaged across channels to give each subject a correlation score with the others. This score measures how close a subject’s brain activity is to the group, with higher scores indicating greater similarity. Subjects were ranked according to these averages. Subjects 14, 18, and 20 were consistently at the top of the list. All three subjects were utilized for DeepConvNet’s baseline training, whereas ATCNet incorporated data solely from subjects 14 and 18. These subjects were chosen to train the base model because their high correlation scores reflect broadly representative neural patterns that are advantageous for capturing features that are common in the population. This approach improves the generalization ability of the model, leading to increased classification accuracy when applied to new or individual subjects.

**Figure 5:**
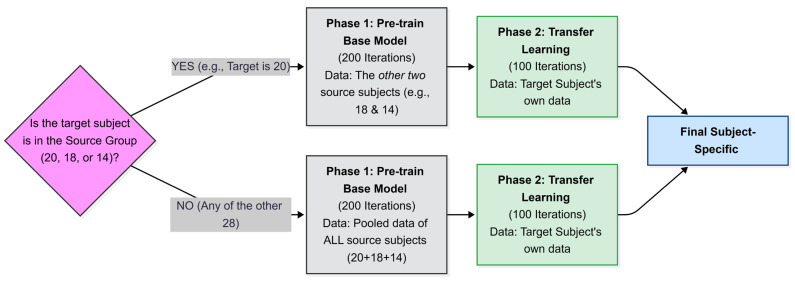
Two-path transfer learning strategy for DeepConvNet. Path selection is determined by the target subject’s membership in the representative source group (14, 18, 20).

The strategy was implemented with minor variations across the three subjects selected for baseline model training. For each subject, the baseline model was trained using data from the remaining two participants in order to evaluate the model’s generalization performance across individuals. For example, the baseline model for subject 20 was trained with data from subjects 14 and 18. Figure 5 provides a visual representation of this procedure for DeepConvNet. For ATCNet, the methodology remains the same, differing only in the inclusion of two base subjects. Transfer learning is uniformly implemented across all experimental configurations in this study. This process allowed the model to learn shared patterns while adapting to the unique characteristics of the target subject. Selecting baseline subjects based on their correlation scores, rather than randomly or by selecting those with high accuracy, provided a solid statistical basis for knowledge transfer [35].

Transfer learning effectively handled data variability, improving classification performance across subjects with different trial numbers and quality. Using the trials of representative subjects (14, 18, and 20) as the basis and preserving all channels made it possible for the DNNs to adapt to both common and individual features. Section 3 emphasizes the value of this transfer learning approach, highlighting the improved generalizability and accuracy.

### 2.4. Signal Augmentation

**Figure 6:**
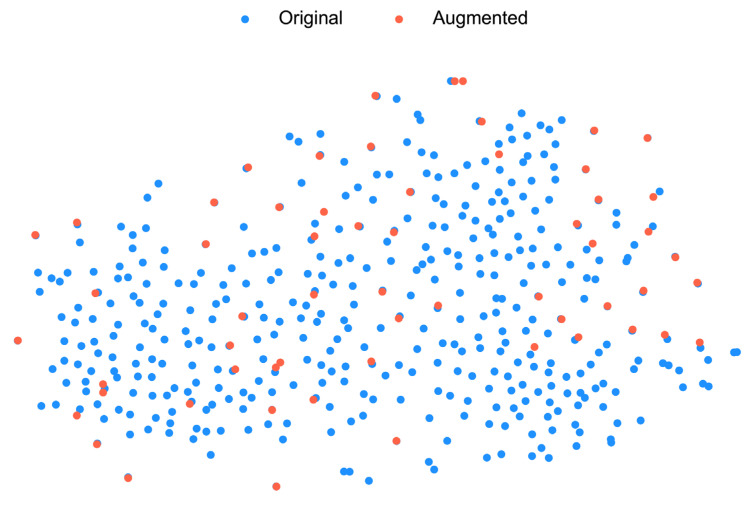
t-SNE projection of original band-power features (blue) versus augmented band-power features (orange), where RF denotes recombination of frequency slices with additive noise to simulate realistic variability.

Data augmentation serves a critical purpose in training deep neural networks, especially when the dataset size is limited, because insufficiently large datasets often fail to capture the full variability of the data distribution [36]. In this study, the t-distributed Stochastic Neighbor Embedding (t-SNE) [37] is used to highlight the sparsity of the original data distribution with data points representing the original and augmented samples (Figure 6). The visualization shows that the augmented samples are well-integrated into the original data clusters, effectively filling the sparse gaps in the feature space and slightly expanding the distribution boundaries without distorting the underlying manifold structure. Furthermore, during cross-validation, 20% of the training data is reserved for validation, reducing the effective training set size. To alleviate these challenges and improve model generalization, augmentation techniques inspired by [38] were used to artificially expand the training dataset and fill these gaps in the data manifold.

Two augmentation methods were combined, namely, Recombination of Frequency Slices (RF) and Gaussian noise addition. The RF method generates synthetic data by recombining frequency components from multiple existing trials, preserving the spectral characteristics of the EEG signals required for their analysis. By adding Gaussian noise to the RF-generated signal, additional variability is accounted for, and the risk of overfitting is reduced [39]. To strike a balance between the benefits of data augmentation and the risk of overfitting, the dataset size was increased by only 15% and augmentation was only applied to the training split during cross-validation. This conservative ratio was determined based on the observation of t-SNE projections to ensure that augmented samples effectively filled the gaps in the data manifold without creating redundant clusters. Recent prominent approaches such as Variational Autoencoders (VAEs) were investigated but were abandoned due to their tendency to produce overly similar samples [40], which can lead to overfitting in scenarios with a limited number of trials.

**Figure 7:**
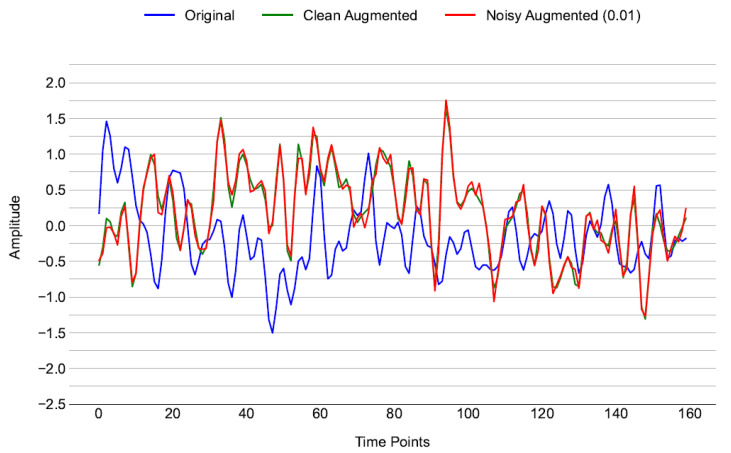
Time-domain signals for selected channels: original signal (blue), clean augmented signal (green), noisy augmented signal with noise level at 0.01 (red).

The effectiveness of these methods is illustrated in Figure 7, which compares an original trial to a generated signal, and shows how the augmented data preserves the key features of the original while generating useful variations. All the following augmentation methods and their associated equations are adapted from George et al. (2022) [38].

#### 2.4.1. Recombination of Frequency Slices (RF)

The RF method generates synthetic data segments by recombining frequency bands from *N* randomly selected source trials (with *N* = 3 in this study). For each channel *c* of the EEG data, the Short Time Fourier Transform (STFT) is computed for the source trials (1). Specifically, the STFT is calculated using a Hann window function with a window length (nperseg) of 64 samples. To maintain temporal continuity and reduce spectral leakage, an overlap of 50% (32 samples) is utilized between consecutive segments. The frequency spectrum is partitioned into *N* disjoint bands, denoted as *B*_1_, *B*_2_, …, *B_N_* (2). In this study, the frequency range of 0.1–45.0 Hz is divided into three specific bands: *B*_1_ (0.1–8.0 Hz) covering delta and theta rhythms, *B*_2_ (8.0–15.0 Hz) focusing on alpha activity, and *B*_3_ (15.0–45.0 Hz) capturing beta and gamma activity. Each band is sourced from a different trial to form a composite STFT. This composite is then transformed back to the time domain using the inverse STFT (ISTFT) to produce the augmented trial (3).

Formally, let *X* represent the input data of shape (trials, channels, samples), and let *x_n_*(*t*) denote the time-domain signal of the *n*-th source trial for a given channel, where *n* = 1, 2, …, *N*. The STFT of each source trial is computed as

STFT{*x_n_*(*t*)} *= X_n_*(*f*, *τ*), (1)

where *f* is the frequency index, *τ* is the time index, and *X_n_*(*f*, *τ*) is the complex-valued STFT of the *n*-th trial.

The composite STFT *X_comp_*(*f*, *τ*) is constructed by assigning each frequency *f* to the corresponding source trial *n* based on the band it belongs to:

*X_comp_*(*f*, *τ*) = *X_n_*(*f*, *τ*), for *f* ∈ *B_n_*, (2)

where *n* ∈ {1, …, *N*} identifies the source trial providing the frequencies in band, *B_n_*. The augmented signal is then obtained via the ISTFT:

*x_aug_*(*t*) = ISTFT{*X_comp_*(*f*, *τ*)} (3)

To ensure the augmented signal matches the original data dimensions, it is padded or truncated as necessary to fit the sample length. The signal is subsequently normalized to maintain an amplitude consistent with the original data, using the root-mean-square (RMS) ratio between the original and augmented signals.

#### 2.4.2. Gaussian Noise Addition

Following the RF augmentation, Gaussian noise is added to the generated data to introduce additional stochastic variability and mitigate overfitting. This step is particularly crucial in cases where the RF-based augmentation might produce similar patterns. The noise level was empirically set to 1% of the signal power to maintain a balance between data regularization and signal integrity. This specific threshold ensures that the added variance prevents the model from memorizing near-identical trials while remaining low enough to avoid distorting essential neurophysiological features, such as frequency oscillations or ERP components. For an augmented trial *x_aug_*(*t*), the noisy signal *x_noisy_*(*t*) is computed as in (4):

*x_noisy_*(*t*) = *x_aug_*(*t*) + *N*(0, *σ*^2^) (4)

where *N*(*0*, *σ*^2^) is zero-mean Gaussian noise with variance *σ*^2^ (5). The variance is determined based on the signal power:

(5) $$\sigma^{2}\;=\;\mathrm{noise\_level}\;\frac{1}{T}\int_{0}^{T}x_{\mathrm{aug}}^{2}\left(t\right)\;dt$$

where *T* is the trial duration and noise_level = 0.01.

### 2.5. Training and Classification

In this study, two deep neural network architectures were used for EEG signal classification: DeepConvNet [34] and ATCNet [41]. DeepConvNet was chosen as it gave the best results in the previous study [3] and deep Convolutional Neural Networks have been the fundamental model of choice for classifying EEG signals. For example, it has shown superior performance in motor imagery tasks compared to traditional methods in studies by Tang et al. (2017) [42] and Robinson et al. (2019) [43]. ATCNet was chosen for its potential to effectively capture sequential data features by utilizing an attention mechanism [44]. The attention mechanism is inspired by the way that humans focus on important details while overlooking less relevant ones. By integrating this approach, the models can focus on the most critical parts of the input data, thereby improving overall performance [41]. Minor adjustments were made to the original architectures of both models to accommodate the input size of the EEG dataset (60 channels and a certain number of samples per trial).

To ensure a robust and subject-specific evaluation of model performance, a 5-fold cross-validation framework was applied independently for each target topic’s dataset. For each participant, the total trial pool was randomly divided into five separate subsets; throughout successive iterations, four of these subsets (80%) formed the training set, while the final subset (20%) was reserved for independent testing.

To facilitate convergence monitoring and reduce the risk of overfitting, an additional nested 20% portion of the training data within each fold was also set aside as a validation set. In configurations using transfer learning, the model was initialized with parameters derived from the baseline architecture. This pre-trained state was then fine-tuned on the target topic’s training data, allowing the system to adapt to its own unique neural models. A strict separation between training and testing was maintained to ensure methodological integrity, preventing data leakage and ensuring that performance metrics accurately reflected the system’s generalization capabilities. This setup was implemented identically for both DeepConvNet and ATCNet with the following basic configurations:

- Model Setup: To make it suitable for the DeepConvNet architecture, the input data were reshaped into the NHWC format (samples, channels, time steps, 1), in which the final dimension (kernels) is typically 1 for EEG data. To adapt the model to the data used in this project, which was sampled at 200 Hz, a kernel size of (1, 8) was used. Additionally, a pool size of (1, 2) was maintained to prevent the temporal dimension from becoming too small for the final convolution layers. For ATCNet, a new axis was added to the input data and transformed into the format (samples, 1, channels, time steps). The model architecture was adjusted to better align with the 200 Hz sampling rate and the 160-sample (0.8 s) epoch length. We reduced the pooling size from 8 to 4 to prevent an excessive loss of temporal resolution; a larger pooling factor at this sampling rate would have overly compressed the signal, potentially hindering the extraction of intricate temporal features. Additionally, the number of sliding windows was set to 3 to strike a balance between effective local feature extraction and reduced data redundancy. Both architectures were compiled using the Sparse Categorical Cross-Entropy loss function and the Adam optimizer.
- Learning Rate Scheduling: To optimize convergence and avoid overfitting, a custom learning rate scheduler was designed by analyzing the training and validation loss curves. The scheduler started with a learning rate of 0.01 for the first 45 epochs, decreased to 0.005 for the next 55 epochs, and decreased to 0.001 for the remaining epochs.
- Training Parameters: 200 epochs with a batch size of 16 and a dropout rate of 0.5 were used to mitigate models memorizing data in training. A model checkpoint callback saved the best model based on the validation accuracy for each fold.
- Transfer Learning Adaptation: When transfer learning was applied, the learning rate was kept constant at 0.001 and the number of epochs was halved to 100 to balance model adaptation and computational efficiency.
- None of the layers were frozen during the transfer learning phase. Instead, the entire network was fine-tuned. This approach was chosen for two main reasons. First, the manageable data size and computational efficiency enabled full model updates without incurring excessive costs. The second important reason is that fine-tuning all layers allows the model to fully adapt to the unique neural characteristics of individual subjects and potentially atypical EEG patterns. By allowing weights to be adjusted across the entire architecture, we aimed to reduce any bias inherited from the pre-training subject pool and increase the model’s generalizability across diverse neural profiles.

Training was implemented in Python using the Keras (3.7.0) library and computations were accelerated on a GPU. The same training protocols were followed, except when transfer learning was applied, in which case the learning rate and number of epochs were adjusted as described. Pre-processing steps (e.g., standardization and power conversion) and signal augmentation (e.g., adding noise at 1% signal strength) were performed before training as described in Section 2.2 and Section 2.4, respectively. To avoid any data leakage, all augmentation operations were re-performed on the new training set defined in each fold in cross-validation; the test set is not included in this process. For the non-augmentation scenarios, DeepConvNet was trained directly on standardized and transformed data using the same 5-fold cross-validation setup.

Performance was assessed by averaging the accuracy of the test set over all five folds, providing a solid measure of the classification capability of each model.

## 3. Results

### 3.1. Comparative Classification Performance

This section compares the performance of the DeepConvNet and ATCNet models when classifying a dataset comprising 31 subjects, along with a subset analysis conducted on 23 subjects as in previous work [24]. Performance metrics include mean accuracy for the 31 and 23 subjects (the latter in brackets) and best accuracy, worst accuracy and standard deviation (STDev) for the subset of 23 subjects (Table 1). The baseline results for Fløtaker et al. [25] and Ludvigsen et al. [24] are cited directly from their respective publications to provide a benchmark against existing literature. The innovative approach proposed in this study is entitled DCN + Transfer Learning + Augmentation. Our findings are summarized in bold in Table 1. We aimed to understand how transfer learning and signal augmentation improved reliability of the system compared to that in [25].

The base DeepConvNet model achieved an average accuracy of 0.765 (0.838) with a STDev of 0.050 for the subset of 23 subjects. Data augmentation improved the average accuracy to 0.773 (0.852). Transfer learning improved accuracies to 0.820 (0.887), and a combination of both techniques gave the highest accuracies of 0.835 (0.90). For ATCNet, the initial accuracies were 0.740 (0.800), while transfer learning improved them to 0.811 (0.874). Fløtaker et al. [3] reported an accuracy of 0.77 (0.847), which outperformed configurations using transfer learning and augmentation. As shown in Table 1, the standard deviation of the accuracy value obtained at different folds in the cross-validation setup decreases. While hyperparameters for the DeepConvNet configurations were kept consistent with those in [25] to ensure a fair comparison, it should be noted that the validation frameworks vary slightly across the literature: Fløtaker et al. [25] utilized a 5-fold cross-validation, while Ludvigsen et al. [24] employed a 10-fold approach. The detailed subject-wise classification results for all 31 participants across all evaluated model configurations are provided in Table A1 of Appendix A.

**Figure 8:**
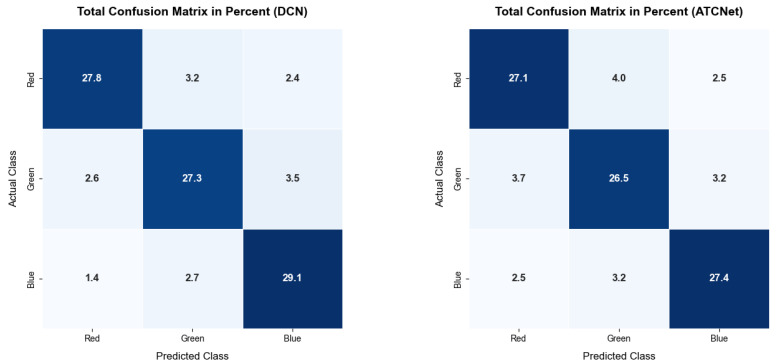
Confusion matrices as percentages for the DeepConvNet (DCN) and Adaptive Temporal Convolutional Network (ATCNet) models, illustrating the classification performance for red, green, and blue color categories across all subjects.

Transfer learning significantly improved average accuracy and also reduced training time from 29.83 s to 16.24 s per fold (for DeepConvNet) by utilizing weights that carry some prior knowledge instead of random weights. It required only 100 epochs, whereas training the base model required 200. The data augmentation applied to DeepConvNet further improved the accuracy by increasing dataset diversity and achieved a better score than ATCNet (Figure 8). Due to the computational demands of ATCNet, augmentation was not applied. The combination of both techniques with DeepConvNet achieved the best results, surpassing those reported in Fløtaker et al. [25]. The deep learning models proposed in this study produced better results than Ludvigsen et al. [24] in terms of both consistency and accuracy for the RGB classification scheme.

Classification analysis for the optimal Deep Convolutional Network (DCN) architecture reveals F1 scores of 0.853, 0.819, and 0.853 for the red, green, and blue categories, respectively, resulting in a macro-average F1 score of 0.842. While these results signify high performance across all chromatic stimuli with no statistically significant variance, the marginally lower F1 score for green is consistent with the elevated error rates observed in specific color pairings. Specifically, green stimuli were misclassified as blue in 3.5% of instances and as red in 2.6% of cases, suggesting a subtle yet systematic difficulty in discriminating green stimuli compared to other wavelengths.

In comparison, the ATCNet model exhibited diminished performance across all metrics, with F1 scores of 0.810 for red, 0.789 for green, and 0.828 for blue, yielding a lower macro-average F1 score of 0.809. Analogous to the DCN model, ATCNet struggled most prominently with the green category, further substantiating the finding that green stimuli present a greater classification challenge within this experimental framework.

### 3.2. Statistical Significance Analysis

To evaluate the statistical significance of the performance improvements and address inter-subject variability, paired Wilcoxon signed-rank tests were conducted for both the full dataset (*N* = 31) and the selected subset (*N* = 23) across three key comparisons.

For the 31-subject dataset, the introduction of transfer learning (DCN vs. DCN + TL) yielded a highly significant improvement in classification accuracy (*p* = 0.000007, r = 0.81). The further addition of signal augmentation (DCN + TL vs. DCN + TL + Aug) also resulted in a statistically significant increase (*p* = 0.000303, r = 0.65), confirming that augmentation provides a unique contribution to model robustness. However, no significant difference was found between the transfer learning-enhanced DCN and ATCNet models (DCN + TL vs. ATCNet + TL, *p* = 0.121501, r = 0.28).

Consistent results were observed for the 23-subject subset. The transition to transfer learning (DCN vs. DCN + TL) was statistically significant (*p* = 0.000135, r = 0.80), as was the enhancement provided by signal augmentation (DCN + TL vs. DCN + TL + Aug, *p* = 0.000419, r = 0.74). Similar to the full dataset, the comparison between DCN + TL and ATCNet + TL did not reach statistical significance (*p* = 0.393957, r = 0.18). These findings provide robust empirical evidence that the proposed combination of transfer learning and signal augmentation significantly enhances EEG-based color decoding performance regardless of the specific deep learning architecture used.

### 3.3. Ablation Analysis: Zero-Shot vs. Adapted Performance

To further investigate the relationship between cross-subject knowledge transfer and individual adaptation, an ablation analysis was performed. The baseline model, pre-trained on the representative subjects 14, 18, and 20, was evaluated on the remaining subjects without any fine-tuning in a Zero-Shot scenario. Subjects 14, 18, and 20 were strictly excluded from these performance evaluation results to ensure an unbiased assessment of cross-subject generalization.

The average accuracy across the remaining subjects was found to be 52.52% for the full group and 57.40% for the 23-subject subset. These results are substantially above the theoretical chance level of 33.3%. This performance indicates that the representative subjects provide a strong generalized feature space for color decoding. The subsequent increase to 82.0% for *N* = 31 and 88.7% for *N* = 23 after fine-tuning, which reached 83.5% for the full group when including signal augmentation, underscores the necessity of adapting these shared features. This adaptation effectively accounts for individual neurophysiological differences, such as unique scalp potential distributions and anatomical variability.

### 3.4. Neurophysiological Analysis and Model Interpretability

This section examines the neurophysiological foundations of color decoding by analyzing both the inherent characteristics of the recorded EEG signals and the feature extraction patterns of the deep learning models. To maintain clarity and provide a concise evaluation, the following analysis focuses on our most robust model configuration: Deep-ConvNet integrated with Transfer Learning. Centering the discussion on this optimal framework ensures that the identified spatio-temporal features and decision-making patterns represent the most reliable neural signatures captured in this study.

#### 3.4.1. Spatio-Temporal Characteristics of Color-Evoked Potentials

**Figure 9:**
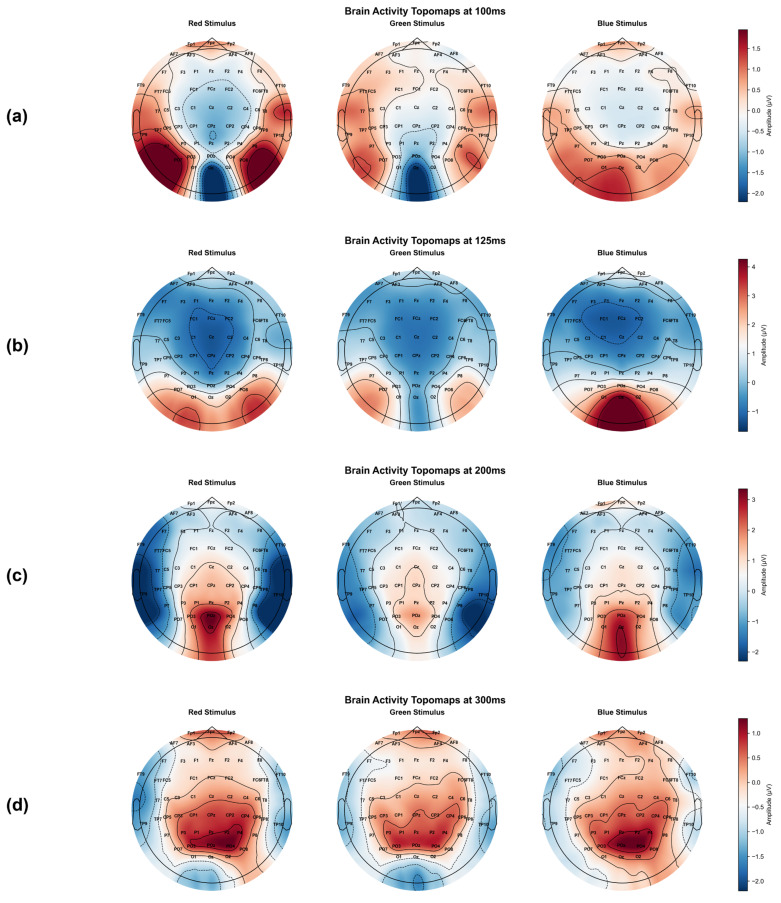
Spatio-temporal topomaps of brain activity for RGB stimuli at (**a**) 100 ms, (**b**) 125 ms, (**c**) 200 ms, and (**d**) 300 ms intervals. The color scale represents neural response amplitude in microvolts (µV). Solid contour lines indicate positive voltage levels, while dashed lines represent negative voltage levels.

The topographical distribution of the grand average event-related potentials (ERPs) for each color stimulus reveals the underlying neurophysiological distinctions within the dataset. Figure 9 presents these brain activity topomaps at 100 ms, 125 ms, 200 ms, and 300 ms to illustrate the raw chromatic information available in the signals. These maps reflect the inherent electrical activity of the brain as a function of color distinctions before any deep learning processing.

- **Initial Sensory Phase (100 ms):** Early sensory activation is clearly visible in the parieto-occipital region, a known cortical signature associated with early visual perception. Red and green stimuli exhibit localized positivity in lateral electrodes such as P7, PO7, P8, and PO8. In contrast, the blue stimulus displays a significantly muted and lower-amplitude early response compared to the other two hues.
- **Peak of Early Visual Processing (125 ms):** This time point marks an intensification of activity at the **occipital pole**. While red stimuli show a strong positive deflection centered around **Oz and O2**, the **blue stimulus** begins to demonstrate a highly focused and high-amplitude central occipital response. The activation for **green** remains the most diffuse and lowest in amplitude among the three stimuli during this window.
- **Wavelength Differentiation (200 ms):** By 200 ms, neural activity for all colors converges toward the central occipital pole. The red stimulus produces a sharp and distinct positive deflection in this area. Green and blue stimuli present broader spatial patterns with less intensity, highlighting the variation in neural geometry across different hues.
- **Cognitive Evaluation and P300 (300 ms):** The 300 ms window identifies the **P300 component**, characterized by a positive deflection over parietal and central areas (CPz, Pz, POz). Notably, the **blue stimulus** elicits the most intense peak amplitude at the center of the scalp. The **red stimulus** remains prominent and spatially extensive, covering a wider area. The **green stimulus** continues to show a lower amplitude and a more fragmented, less coherent distribution compared to red and blue.

The systematic variation in these potentials confirms that the recorded EEG signals contain robust and discriminable chromatic signatures. These inherent spatio-temporal features in the data provide the fundamental “neural descriptors” that facilitate high-accuracy classification in subsequent stages of the study.

**Figure 10:**
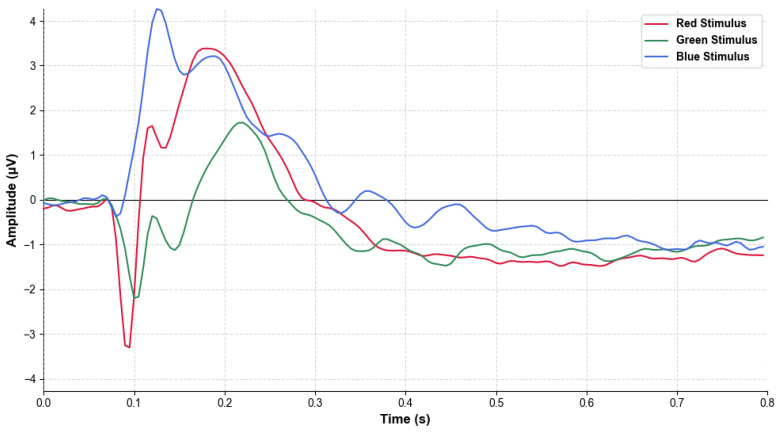
Grand average Event-Related Potential (ERP) waveforms at channel Oz for red, green, and blue color stimuli. The plot illustrates neural response amplitudes (µV) within the 0.0 to 0.8 s post-stimulus time window.

To quantitatively validate the neurophysiological distinctions observed in the topographical maps, a repeated-measures ANOVA was conducted on the peak amplitudes and latencies of the P100, P200, and P300 components recorded from the Oz channel. This analysis provides a statistical basis for the neural descriptors that facilitate high-accuracy classification. The resulting ERP waveforms, representing the average of all 31 subjects, are illustrated in Figure 10, providing a statistical and visual basis for the “neural descriptors” that facilitate high-accuracy classification.

The statistical analysis revealed highly significant differences across color conditions. For the P100 component, the Green–Blue (G-B) comparison showed a substantial effect size in amplitude (Cohen’s d = −1.6151, *p_adj* < 0.001). The P200 component exhibited the most pronounced differences, with Green–Red (G-R) and Green–Blue (G-B) comparisons yielding large effect sizes for amplitude (d = −1.4320 and d = −1.0481, respectively). These findings suggest that different wavelengths trigger distinct automatic attention processes.

Furthermore, these statistical results offer a clear explanation for the lower F1 score of the green category. The consistently lower peak amplitudes and more fragmented neural patterns observed for green stimuli indicate a less robust and less distinct neural signature compared to red and blue. This reduced signal coherence likely makes it more difficult for the deep learning models to establish stable feature boundaries for green, leading to systematic misclassifications and marginally higher error rates observed in the confusion matrices.

#### 3.4.2. Model Interpretability and Feature Validation

**Figure 11:**
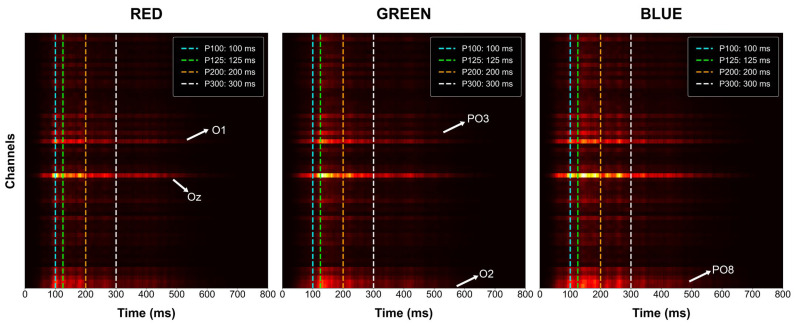
Spatio-temporal saliency maps of the DeepConvNet model. Heatmaps show channel importance for RGB categories relative to ERP latencies (dashed lines). Arrows highlight key electrodes (e.g., Oz, PO8) prioritized by the model.

To validate that the high classification performance of the DeepConvNet (DCN) model is grounded in neurophysiologically relevant features rather than artifacts or noise, saliency map analysis was performed on the pre-trained baseline architecture. This interpretability approach identifies the specific spatio-temporal features within the EEG signal that contributes most significantly to the model’s decision-making process for each color category. The grand average saliency maps, illustrated in Figure 11, represent the gradient-based importance of each channel across the 800 ms post-stimulus window, with overlaid markers for the previously identified P100, P125, P200, and P300 components.

The spatial distribution of the saliency maps demonstrates a high degree of alignment with the primary and secondary visual processing areas. For all three color categories, the model primarily prioritizes signals from the occipital and parieto-occipital electrodes, which is consistent with the cortical signatures of visual perception. Specifically, for the red stimulus, the model exhibits concentrated importance at the Oz and O1 channels, with peak saliency occurring between the 100 ms and 200 ms windows. This temporal focus directly corresponds to the early sensory activation and wavelength differentiation phases identified in the ERP analysis.

The blue stimulus saliency map reveals a distinct reliance on the Oz and PO8 channels, with high-importance clusters localized around the 100 ms and 300 ms marks. This pattern aligns with the neurophysiological observation that the blue stimulus elicits a focused central occipital response during early processing and a particularly intense peak amplitude at the center of the scalp during the P300 phase. In contrast, the saliency map for the green stimulus displays a more diffuse pattern of importance across the PO3, Oz, and O2 channels. While it captures relevant activity around the 125 ms window, the lower signal coherence and more fragmented distribution of green-evoked potentials—as discussed in the neurophysiological analysis—are reflected in these more spread-out saliency clusters.

The systematic overlap between the high-saliency regions and the statistically significant ERP components (P100, P200, and P300) confirms that the deep learning model has successfully learned to exploit robust chromatic signatures. By focusing on the temporal windows where wavelength differentiation is most pronounced, such as the 200 ms interval, the DCN architecture effectively captures the automatic attention processes triggered by different colors. This convergence between model-derived features and established neurophysiological markers provides strong empirical evidence for the reliability and validity of the proposed decoding methodology.

**Figure 12:**
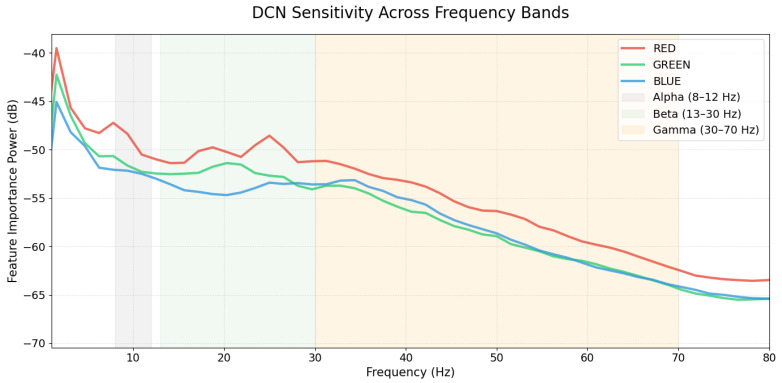
Frequency-domain sensitivity of the DeepConvNet (DCN) model. The plot illustrates the spectral power (dB) of saliency maps across red, green, and blue stimuli. Shaded regions represent the Alpha (8–12 Hz), Beta (13–30 Hz), and Gamma (30–70 Hz) bands, highlighting the model’s reliance on specific neural oscillations for chromatic decoding.

Figure 12 shows the interaction between different frequency bands. A clear **inverse relationship** is observed: feature importance is highest at low frequencies (0–10 Hz) and steadily declines as the frequency increases toward 80 Hz. This indicates that the model relies more heavily on low-frequency components for its predictions than on high-frequency signals.

Among the three groups, the **RED** channel consistently demonstrates the highest sensitivity across the entire spectrum. It shows prominent peaks within the **Alpha (8–12 Hz)** and **Beta (13–30 Hz)** bands, particularly around 10 Hz and 25 Hz. In contrast, the GREEN and BLUE groups exhibit lower and more similar importance levels, eventually converging in the **Gamma band (30–70 Hz)** where their influence reaches a minimum.

## 4. Discussion

Given the complex nature of EEG signals, advanced signal-processing techniques and robust classification methods are required to interpret the underlying neural mechanisms elicited by visual stimuli. Studies that attempt to decode visual stimuli containing complex objects typically report lower classification accuracies [12] compared with those using simple shapes or color-based stimuli [45].

This research investigated the effect of transfer learning and data augmentation on the classification of EEG signals altered by RGB color stimuli using DNN architectures in electrode space. The results show that these methods significantly improve the accuracy and consistency of classification across subjects. Transfer learning successfully mitigated challenges such as cross-subject variability and limited data to improve performance and reduce training time using fewer training iterations. Data augmentation slightly improved accuracy by increasing dataset diversity, benefiting DeepConvNet. The combined techniques proposed in this study achieved the highest accuracy rates, emphasizing the effectiveness of these methods. Statistical analysis confirmed that these improvements are highly significant, with transfer learning (*p* < 0.001) and signal augmentation (*p* < 0.001) providing robust contributions to model accuracy. Furthermore, the ablation analysis demonstrated a substantial ‘adaptation gap’: while the base model achieved a Zero-Shot accuracy of 52.5%—well above the chance level of 33.3%—fine-tuning was essential to reach the peak accuracy over 80%. This indicates that while representative subjects provide a strong generalized feature space, individual adaptation is critical for accounting for subject-specific anatomical and neurophysiological variability.

We also observed some limitations. ATCNet has a more complex structure than DeepConvNet. This computational complexity limits its use with augmentation, and the architecture needs to be optimized to take full advantage of these techniques. Examination of the confusion matrices and F1 scores reveals that the green category is harder to decode (F1 = 0.819) compared to red and blue. This finding is directly supported by the neurophysiological analysis in Section 3.4.1, which identified significantly lower peak amplitudes and less coherent spatial distributions for green-evoked potentials. This suggests that the lower classification performance is not merely a model artifact but a reflection of the weaker neural signature inherent to that specific wavelength.

Other classification methods were applied to this dataset as well as the one presented in this paper (Table 1). An equal feature extraction method in combination with a tangent space Riemannian classifier performed marginally poorer with an average accuracy of 74.4% [24]. The best result that Fløtaker et al. [25] reported with DeepConvNet was an average classification accuracy of 77% for RGB color classification and a peak accuracy of 96% for the best performing subject. In comparison, the peak accuracy achieved in this study was found to be 97%, and the average classification accuracy was found to be 83.5%, indicating an improvement over previous results reported in the related literature.

Energy, fractal, and statistical features were also extracted and used in combination with classifiers such as LDAs with shrinkage, RF, and SVM [24]. The accuracy of 83.5% obtained when classifying RGB-colors in this study is higher than the average accuracies obtained in [24,46,47], which were 74.4%, 46%, and 70.2%, respectively.

Crucially, the interpretability analysis in Section 3.4 validates that the DeepConvNet model relies on neurophysiologically grounded features rather than noise. Saliency maps revealed that the model prioritizes occipital and parieto-occipital electrodes (Oz, PO8) within the 100–300 ms time window, aligning perfectly with the P100, P200, and P300 ERP components identified in the signal analysis. Additionally, frequency domain sensitivity analysis (Figure 12) demonstrated a clear inverse relationship between frequency and feature importance: the model places the highest weight on low-frequency Alpha (8–12 Hz) and Beta (13–30 Hz) oscillations, while importance steadily declines toward the Gamma band. This confirms that the model actively filters out high-frequency noise and focuses on the neural bands most associated with visual processing.

Studies employing machine-learning-based classification typically incorporate features extracted from EEG time-series data. From a physiological perspective, alpha-band oscillations over the parieto-occipital region are often computed, as they represent a cortical signature associated with both visual imagery and visual perception [48]. However, in [49], changes in beta and theta oscillations in the occipital region and changes in phase consistency in the prefrontal area were observed. Another relevant physiological marker is the P300 component of the event-related potential (ERP), characterized by a positive deflection occurring approximately 300 milliseconds after stimulus presentation [50]. In addition to the amplitudes of the ERP components, their latencies were also found to be related to the perceived color changes [51].

The findings of the study suggest that transfer learning and data augmentation are effective strategies for improving EEG signal classification using deep learning. It enhances the reliability of solutions while helping them to remain scalable for applications such as brain–computer interfaces. In this direction, future research should optimize attention mechanisms in models such as ATCNet to reduce computational demands. Exploring cross-subject transfer learning to improve applicability and performance in diverse contexts will also be beneficial. Furthermore, while this study focused on a color decoding task, future work should evaluate the proposed strategy on more complex EEG tasks and large-scale publicly available benchmark datasets, such as EEG-Things2, to further validate its generalizability across diverse experimental paradigms. Finally, since the saliency map analysis (Figure 11) confirmed that the most discriminative features are spatially localized to the occipital pole, future research should extend this approach to source space analysis. Transitioning from electrode space to source space could further resolve the spatial overlap observed in the green-stimulus potential, potentially mitigating the lower classification performance observed for that category.

## 5. Conclusions

This research highlights the potential of integrating transfer learning and signal augmentation to enhance EEG-based RGB color classification. By utilizing deep learning architectures such as DeepConvNet and ATCNet, the models were first trained on subjects with representative brain patterns and then adapted to new individuals, effectively capturing personal differences in EEG signals. Augmentation methods like frequency slice recombination and the addition of Gaussian noise contributed to increased data diversity, which in turn improved the models’ robustness. Achieving an accuracy rate of 83.5% across 31 participants, the proposed approach demonstrates statistically significant advantages over traditional methods. Furthermore, saliency map analysis confirms that the model’s high performance is driven by plausible neurophysiological features—specifically P100 and P200 components in the occipital region—rather than artifacts. These results affirm the value of cross-subject learning and synthetic data enhancement in overcoming the limitations imposed by limited and variable EEG datasets, supporting more reliable performance in future BCI and neural decoding applications.

## Figures and Tables

**Table 1:** Comparison of classification performance metrics between the proposed DCN + Transfer Learning + Augmentation-based combined method and existing approaches reported in the literature. The values outside parentheses are the mean accuracy over all 31 subjects, and values inside parentheses are the mean accuracy for the selected 23-subject subset; for this subset the best and worst accuracies and the standard deviation are also summarized.

Model Configuration	Accuracy	Best	Worst	STDev
DCN	0.765 (0.838)	0.960	0.680	0.048
DCN + Augmentation	0.773 (0.852)	0.960	0.740	0.041
DCN + Transfer Learning	0.820 (0.887)	0.970	0.800	0.037
**DCN + Transfer Learning + Augmentation**	**0.835 (0.900)**	**0.970**	**0.810**	**0.034**
ATCNet	0.740 (0.800)	0.950	0.690	0.049
ATCNet + Transfer Learning	0.811 (0.874)	0.960	0.780	0.034
Fløtaker et al.	0.774 (0.847)	0.960	0.660	0.040
Ludvigsen et al.	NA (0.774)	0.930	0.540	0.075

## Data Availability

The raw data supporting the conclusions of this article will be made available by the NTNU on request. More details regarding the data are published in [25].

## Abbreviations

The following abbreviations are used in this manuscript:

EEG	Electroencephalography
BCI	Brain Computer Interface
RGB	Red green blue
CNN	Convolutional neural network
DCN	DeepConvNet
ATCNet	Agile Temporal Convolutional Network
FgMDM	the Fisher geodesic Minimum Distance to the Mean
VEP	Visual evoked potential
LDA	Linear Discriminant Analysis
CIELAB	The International Commission on Illumination Lab (Commission internationale de l’éclairage)
MRI	Magnetic Resonance Imaging
EOG	Electrooculography
DNN	Deep neural network
t-SNE	t-distributed Stochastic Neighbor Embedding
RF	Recombination of Frequency slices
VAE	Variational Autoencoder
STFT	Short-Time Fourier Transform
ISTFT	Inverse short-time Fourier transform
RMS	Root mean square
SSVEP	Steady-state visually evoked potential
TL	Transfer learning
Aug	Augmentation

## Appendix A

Table A1 provides the detailed classification performance for each individual subject across all evaluated model configurations. While the main text reports aggregate metrics, this table offers a granular view of the performance variability among the 31 participants. The results for Ludvigsen et al. were obtained from the original publication, whereas the results for Fløtaker et al. were sourced from the more detailed version of their study as reported in [52]. The distinction between the full dataset (AVG) and the selected 23-subject subset (AVG*) is maintained here to provide full transparency regarding the data exclusion criteria and its impact on the overall model evaluation.

**Table A1:** Detailed classification accuracy for each subject across all model configurations. The upper section contains the 23 subjects included in the primary subset (AVG*), while the lower section (below the midrule) contains the subjects excluded from the subset. Best performance for each subject is often achieved by the DCN + TL + Augmentation configuration.

Subject	Ludvigsen et al.	Fløtaker et al.	DCN	DCN + Aug	DCN + TL	DCN + TL + Aug	ATCNet	ATCNet + TL
13	0.92	0.96	0.96	0.96	0.97	0.97	0.95	0.96
21	0.90	0.93	0.95	0.95	0.94	0.94	0.93	0.93
18	0.75	0.92	0.90	0.89	0.93	0.95	0.90	0.91
29	0.83	0.92	0.93	0.92	0.94	0.94	0.69	0.90
6	0.85	0.90	0.93	0.93	0.96	0.97	0.92	0.96
20	0.71	0.90	0.85	0.86	0.89	0.90	0.84	0.86
14	0.93	0.89	0.93	0.93	0.94	0.93	0.91	0.90
19	0.76	0.89	0.85	0.86	0.96	0.97	0.84	0.91
24	0.76	0.89	0.87	0.89	0.93	0.94	0.85	0.92
2	0.92	0.87	0.89	0.90	0.94	0.93	0.77	0.91
30	0.82	0.87	0.89	0.84	0.89	0.89	0.76	0.82
26	0.80	0.86	0.83	0.84	0.87	0.87	0.81	0.84
7	0.78	0.85	0.88	0.90	0.91	0.94	0.93	0.92
8	0.65	0.84	0.81	0.85	0.83	0.91	0.69	0.78
23	0.75	0.84	0.84	0.82	0.87	0.89	0.81	0.88
11	0.75	0.83	0.80	0.81	0.85	0.87	0.71	0.78
5	0.73	0.82	0.82	0.85	0.87	0.87	0.75	0.86
15	0.55	0.82	0.75	0.77	0.80	0.82	0.69	0.92
3	0.64	0.80	0.69	0.76	0.85	0.89	0.70	0.88
25	0.67	0.75	0.74	0.79	0.80	0.83	0.69	0.82
31	0.54	0.74	0.75	0.77	0.83	0.83	0.80	0.83
16	0.54	0.72	0.73	0.74	0.82	0.81	0.71	0.84
28	0.57	0.66	0.68	0.77	0.82	0.81	0.76	0.79
22	–	0.68	0.65	0.61	0.79	0.79	0.65	0.72
4	–	0.66	0.76	0.71	0.81	0.83	0.75	0.80
27	–	0.65	0.52	0.51	0.58	0.61	0.57	0.68
12	–	0.58	0.42	0.44	0.47	0.47	0.41	0.45
9	–	0.51	0.53	0.58	0.72	0.76	0.59	0.69
17	–	0.51	0.51	0.51	0.50	0.53	0.47	0.54
1	–	0.47	0.48	0.46	0.59	0.58	0.50	0.52
10	–	0.46	0.59	0.53	0.58	0.63	0.60	0.65
AVG	0.744	0.774	0.765	0.773	0.821	**0.835**	0.740	0.812
AVG*	0.744	0.847	0.838	0.852	0.887	**0.900**	0.800	0.874
MIN	0.540	0.660	0.680	0.740	0.800	0.810	0.690	0.780
MAX	0.930	0.960	0.960	0.960	0.970	0.970	0.950	0.960
